# Prevalence of hyperfunctioning thyroid nodules among those in need of fine needle aspiration cytology according to ATA 2015, EU-TIRADS, and ACR-TIRADS

**DOI:** 10.1007/s00259-020-04740-y

**Published:** 2020-03-09

**Authors:** Benjamin Noto, Maria Eveslage, Michaela Pixberg, José Manuel Gonzalez Carvalho, Michael Schäfers, Burkhard Riemann, Peter Kies

**Affiliations:** 1 Department of Nuclear Medicine, University Hospital of Münster, Albert-Schweitzer-Campus 1, 48149 Münster, Germany; 2 Institute of Biostatistics and Clinical Research, University of Münster, Münster, Germany

**Keywords:** TIRADS, Thyroid scintigraphy, Thyroid nodules, Sonography, Hyperfunctional thyroid nodules

## Abstract

**Purpose:**

Given the large number of patients with thyroid nodules, improvement of the specificity of current ultrasound-based thyroid nodule classification systems (ATA, EU-TIRADS, and ACR-TIRADS) is warranted to reduce the number of diagnostic thyroidectomies. Thyroid scintigraphy has been shown to demonstrate hyperfunctional nodules, associated with a low malignancy risk, in euthyroid patients. However, it is not known if thyroid scintigraphy could improve specificity of current classification systems. The aim of this study, therefore, was to determine the frequency of hyperfunctional nodules among those nodules in need of fine needle aspiration cytology (FNA) according to current classification systems and to test if nodule functional status is associated with sonographic features.

**Methods:**

Five hundred sixty-six euthyroid patients (TSH 0.55–4.20 μU/ml) presenting for thyroid nodule workup including thyroid sonography and scintigraphy at our department between 09/2013 and 02/2018 were included in this retrospective study. All nodules > 10 mm were classified according to ATA, EU-TIRADS, and ACR-TIRADS and correlated to their functional status as assessed by ^99m^Tc-pertechnetate scintigraphy.

**Results:**

Ultrasound detected 1029 thyroid nodules ≥ 10 mm, including 545 nodules ≥ 15 mm. Prevalence of hyperfunctional nodules among those with recommendation for FNA according to ATA 2015, EU-TIRADS, and ACR-TIRADS was 6.4%, 6.9%, and 6.5% for nodules ≥ 10 mm and 7.2%, 7.6%, and 7.5% only considering nodules ≥ 15 mm. No sonographic feature was correlated to hyperfunctionality of nodules.

**Conclusion:**

In euthyroid patients, thyroid scintigraphy demonstrates hyperfunctionality, which cannot be predicted by ultrasound, in up to 6.9% of nodules in need of FNA according to ultrasound-based classifications. Given the known low risk of malignancy in hyperfunctional nodules, thyroid scintigraphy can lower the frequency of fine needle aspirations and—potentially—the frequency of diagnostic hemithyroidectomies in euthyroid patients.

## Introduction

Thyroid nodules are a common diagnostic challenge occurring in up to 68% of randomly selected individuals [1]. This is especially true in iodine-deficient regions since the prevalence of nodular goiter is directly related to the degree of iodine deficiency [2]. While some nodules harbor malignancy, the vast majority are benign [3].

Currently, the two primary methods used for workup of thyroid nodules are sonography and fine needle aspiration cytology (FNA). Commonly used classification systems for nodule workup are proposed by the American Thyroid Association (ATA 2015) [4], the European Thyroid Association (EU-TIRADS) [5], and the American College of Radiology (ACR-TIRADS) [6]. All classification systems estimate the risk of malignancy of a thyroid nodule based on ultrasound features. Depending on the size of the nodule and the number of suspicious ultrasound features, FNA is recommended [7].

While the implementation of standardized ultrasound-based classification systems has led to an improvement in the assessment of thyroid nodules, resulting specificities and accuracies are still rather low. In a recent study evaluating the ACR-TIRADS classification system, a high sensitivity of 92% was demonstrated, but specificity and accuracy were only 44% and 52% respectively [7]. This low specificity combined with a high prevalence of thyroid nodules results in a high rate of fine needle aspirations and—potentially—unnecessary thyroidectomies [8]. A further improvement in thyroid nodule evaluation is therefore warranted.

In contrast to ultrasound, thyroid scintigraphy assesses the functional status of thyroid nodules. Its main application is the workup of hyperthyroidism [9]. Diagnostic thyroid scintigraphy is typically performed with ^99m^Tc-pertechnetate, which is taken up into the follicular thyroid cell by the sodium/iodine symporter (NIS) [10]. Hyperfunctioning thyroid tissue exhibits an increased expression of the NIS and therefore appears with increased tracer uptake on thyroid scintigraphy. Hyperfunctional thyroid nodules are very unlikely to harbor malignancy, i.e., the negative predictive value of thyroid scintigraphy for malignancy is very high [11–13].

Interestingly, in recent meta-analyses published by Treglia et al., up to 50% of hyperfunctional nodules occurred in patients with normal TSH levels [14, 15]. However, it has not been systematically studied if thyroid scintigraphy could have an incremental diagnostic value in euthyroid patients over ultrasound-based classification. If a nodule that is in need of FNA according to ultrasound classification appears hyperfunctional in thyroid scintigraphy, the estimated risk of malignancy is low, and FNA and—potentially—diagnostic hemithyroidectomy can be avoided [16–18].

Recently, Schenke et al. [19] investigated if TIRADS according to Kwak [20] identifies hyperfunctional thyroid nodules as non-suspicious. The study included 615 autonomous thyroid nodules in 582 patients with and without hyperthyroidism. Eight percent of the investigated autonomous nodules were classified as suspicious by TIRADS, indicating a complementary value of thyroid scintigraphy.

However, the prevalence of hyperfunctional nodules in euthyroid patients and its relationship to ultrasound-based classification has not been sufficiently studied. To analyze the potential incremental value of thyroid scintigraphy in euthyroid patients, this study aimed at analyzing the frequency of hyperfunctional nodules in a large cohort of euthyroid patients and its relation to state-of-the-art ultrasound-based classification systems.

## Methods

### Patients and study design

For this retrospective analysis, a cohort of euthyroid patients (TSH 0.55–4.20 μU/ml), coming from a population with past and present iodine deficiency, was enrolled which presented at our department for the workup of thyroid nodules (normal range for TSH 0.55–4.78 μU/ml up to February 2017, afterwards 0.27–4.20 μU/ml according to a new assay) between September 2013 and April 2018. Patients with a ^99m^Tc-pertechnetate thyroid scintigraphy (routinely performed for thyroid nodules > 10 mm according to national guidelines [21]), without previous radioiodine therapy, thyrostatic drugs, or levothyroxine medication were enrolled. When available in the patient records, results of fine needle aspiration cytology and histopathology were noted. FNA results were classified according to the Royal College of Pathologists Guidance on the reporting of thyroid cytology specimens [22].

The study protocol was approved by the ethics committee of the University of Münster (2019-237-f-S) and performed in accordance with the ethical standards as laid down in the 1964 Declaration of Helsinki and its later amendments.

### Imaging

Ultrasound of the thyroid gland was performed using either a Philips iU22, a Siemens Acuson NX3 Elite, or a Siemens Acuson S1000 with a linear probe with a frequency of 5–10 MHz. Volume of the thyroid gland, nodule size in all three dimensions, and nodule position within the thyroid were documented. Thyroid scintigraphy was acquired according to German guidelines 10–25 min post injection of 70 MBq ^99m^Tc-pertechnetate with a MIE Scintron (LEHR collimation, matrix 128 × 128; acquisition time 10 min) [21].

Sonographic and scintigraphic images were retrospectively evaluated by either one of two nuclear medicine physicians with extensive experience in thyroid medicine and thyroid ultrasound (more than 4000 thyroid ultrasound examinations performed every year in the department). Images were viewed using a commercial picture archiving and communication system (General Electric Centricity PACS RA1000). Thyroid nodules larger than 10 mm were classified according to ATA 2015, EU-TIRADS, and ACR-TIRADS criteria and all sonographic features required for the respective classification system (composition, echogenicity, shape, margin, calcifications) were noted for further analysis (Table 1). Nodule classification according to ATA 2015, EU-TURADS, and ACR-TIRADS was performed without knowledge of the scintigraphic appearance of the nodule.

**Table 1:** Sonographic characteristics of evaluated thyroid nodules (*n* = 1029)

Composition	Cystic or completely cystic (7.6%)	Spongiform (12.7%)	Mixed cystic and solid (31.8%)	Solid or almost completely solid (47.9%)	
Echogenicity	Anechoic (5.9%)	Very hypoechoic (1.5%)	Hypoechoic (16.4%)	Hyper- or isoechoic (76.2%)	
Shape	Wider than tall (97.2%)		Taller than wide (2.8%)		
Margin	Smooth (93.9%)	Ill-defined (4.2%)	Lobulated or irregular (1.4%)	Extra-thyroidal Extension (0.6%)	
Hyperechoic components	None (90.7%)	Large comet tail artifacts (1.9%)	Macro calcifications (3.3%)	Peripheral rim calcification (0.8%)	Punctate echogenic foci (3.3%)

Sonographically detected nodules were subsequently also classified by their ^99m^Tc-pertechnetate uptake in the thyroid scintigraphy as either *hypofunctional* (below the level of the surrounding thyroid tissue), *indeterminate* (in the level of the surrounding thyroid tissue), or *hyperfunctional* (above the level of the surrounding thyroid tissue).

### Data and statistical analysis

Analysis was performed with commercially available software (IBM® SPSS® Statistics 24, IBM Corporation, Somers, NY, USA and SAS® software, version 9.4 for Windows, SAS Institute, Cary, NC, USA). Thyroid nodules are clustered within patients since in many cases multiple nodules per patient are observed. To account for the resulting intra-individual correlation, nominal variables were tested for their association with hyperfunctional thyroid nodules by using generalized linear mixed models. A random intercept for the patient was included to account for intra-patient correlation. Odds ratios, corresponding 95% confidence intervals, and *p* values were calculated in SAS using proc glimmix with logit link.

Due to outliers, the non-parametric Mann-Whitney *U* test was used to compare age and TSH values of patients with or without hyperfunctional thyroid nodules. Also due to outliers, the non-parametric Kruskal-Wallis *H* test was used to compare nodule size between hypofunctional, indeterminate, and hyperfunctional nodules in thyroid scintigraphy. Dunn’s procedure with a Bonferroni correction for multiple comparisons was used for post hoc analysis. All reported *p* values are two-sided. A value of *p* < 0.05 was considered statistically significant.

## Results

### Patient characteristics

Five hundred sixty-six patients fulfilled the above inclusion criteria. Sixty-five percent were female and 35% male. Median age was 54.0 years (range 13–87 years). Median TSH was 1.20 μU/ml (range 0.55–4.16 μU/ml).

### Imaging results

Overall, 1029 nodules larger than 10 mm were detected with an average of 1.8 nodules per patient (range 1–9) and a diameter of 17.8 ± 8.7 mm (mean ± standard deviation). Sonographic features of the nodules are listed in Table 1.

**Fig. 1:**
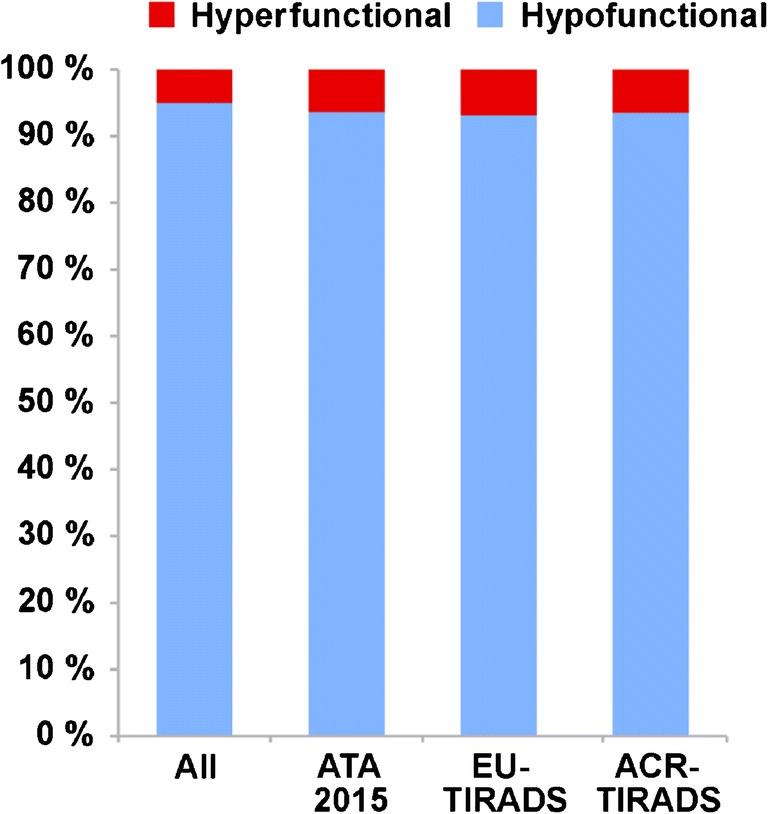
Prevalence of hyperfunctional nodules among all nodules (all), and among those nodules recommended for FNA according to different ultrasound classification systems

On ^99m^Tc-pertechnetate scintigraphy, 353 thyroid nodules (34.3%) were hypofunctional, 625 (60.7%) indeterminate, and 51 (5.0%) hyperfunctional. Hyperfunctional nodules were found in 47 (8.3%) patients (Fig. 1).

### Classification of thyroid nodules and correlation to scintigraphy results

**Fig. 2:**
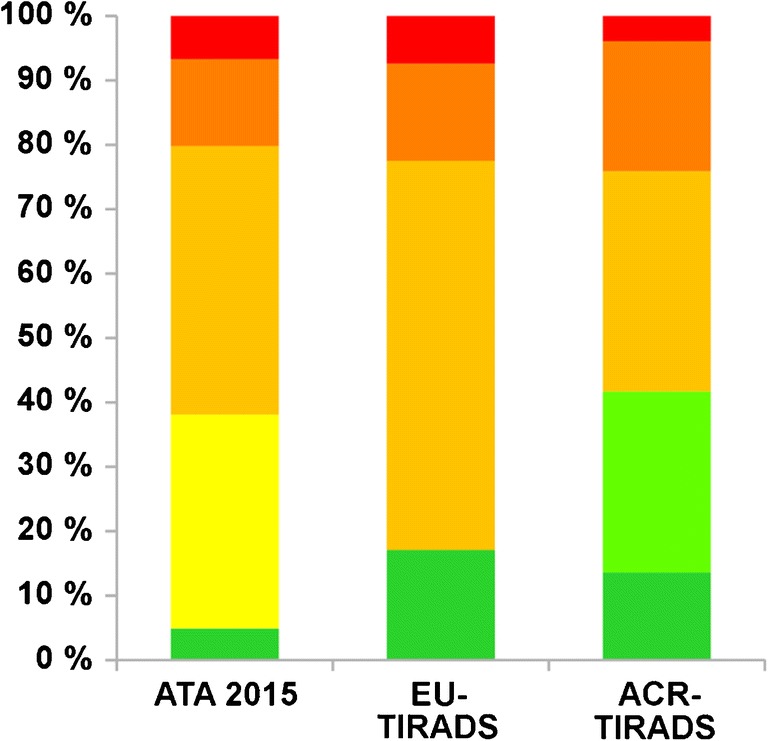
Sonographic classification of all 1029 thyroid nodules using different classification schemes. ATA 2015: benign (dark green), very low suspicion (yellow), low suspicion (light orange), intermediate suspicion (dark orange), high suspicion (red). EU-TIRADS: benign/EU-TIRADS 2 (dark green), low risk/EU-TIRADS 3 (light orange), intermediate risk/EU-TIRADS 4 (dark orange), high risk/EU-TIRADS 5 (red). ACR-TIRADS: benign/TR1 (dark green), not suspicious/TR 2 (light green), mildly suspicious/TR 3 (light orange), moderately suspicious/TR 4 (dark orange), highly suspicious/TR 5 (red)

**Fig. 3:**
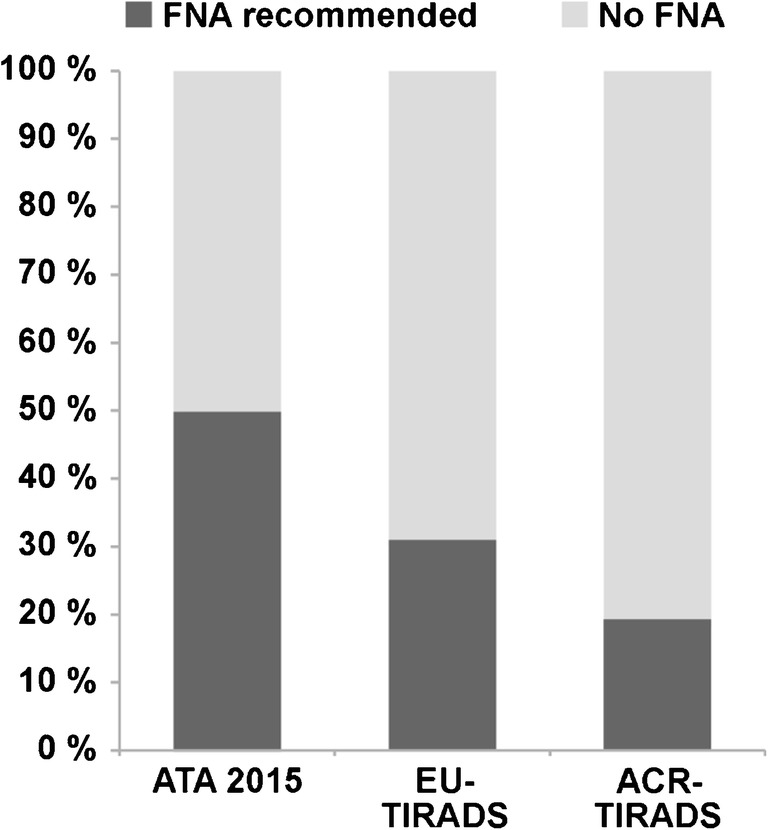
Proportion of nodules (out of the entire 1257 nodules) for which FNA is recommended according to different ultrasound classification schemes

**Fig. 4:**
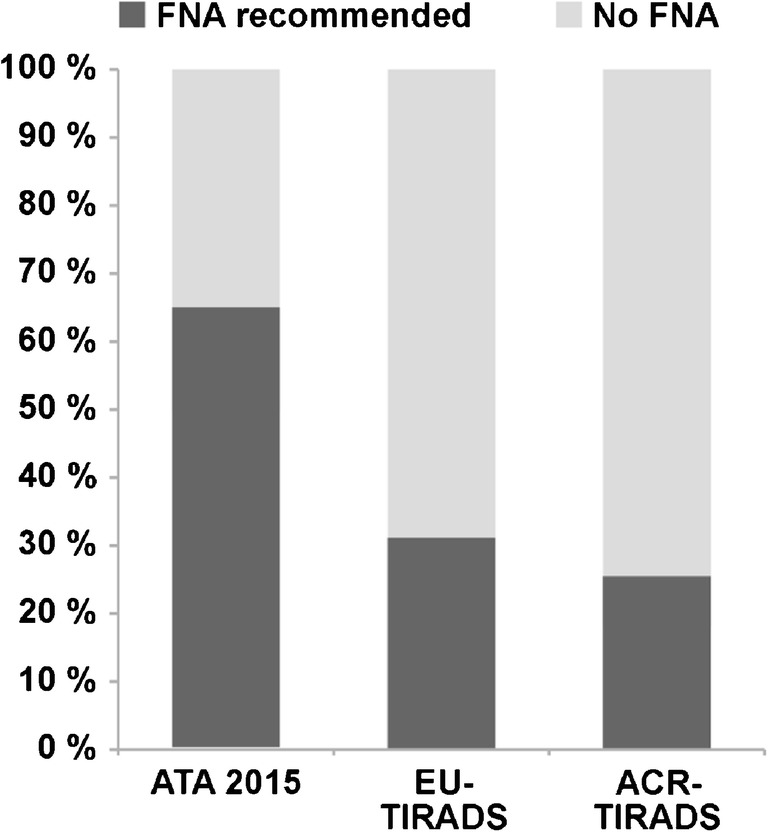
Proportion of the 61 hyperfunctional nodules for which FNA is recommended according to different ultrasound classification systems

Classification of all evaluated nodules and hyperfunctional nodules according to the three different ultrasound-based classification systems is described in Table 2 and illustrated in Fig. 2. The proportion of nodules for which FNA was recommended was highest for ATA 2015 (50.0%) and lowest for ACR-TIRADS (19.3%) (Fig. 3). Also, the proportion of the 51 hyperfunctional nodules for which FNA was recommended was highest for ATA 2015 (64.7%) followed by EU- (43.1%) and ACR-TIRADS (25.5%) (Fig. 4). The lesion-based prevalence of hyperfunctional nodules under those recommended for FNA considering all nodules was 6.4%, 6.9%, and 6.5% for ATA, EU-TIRADS, and ACR-TIRADS. Considering only nodules ≥ 15 mm, the prevalence was 7.2%, 7.6%, and 7.5% for ATA, EU-TIRADS, and ACR-TIRADS (Table 3). The patient-based prevalence of hyperfunctional nodules is described in Table 3.

**Table 2:** Classification of all evaluated thyroid nodules (*n* = 1257) and hyperfunctional nodules (*n* = 61) according to ATA 2015, EU-TIRADS, and ACR-TIRADS

Category	All nodules	Hyperfunctional nodules
ATA 2015		
Benign	4.9%	0%
Very low suspicion	33.2%	39.2%
Low suspicion	41.7%	29.4%
Intermediate suspicion	13.5%	25.5%
High suspicion	6.7%	5.9%
EU-TIRADS		
Benign (EU-TIRADS 2)	17.0%	19.6%
Low risk (EU-TIRADS 3)	60.4%	49.0%
Intermediate risk (EU-TIRADS 4)	15.1%	23.5%
High risk (EU-TIRADS 5)	7.4%	7.8%
ACR-TIRADS		
Benign (TR1)	13.6%	17.6%
Not suspicious (TR2)	28.1%	17.6%
Mildly suspicious (TR3)	34.2%	33.3%
Moderately suspicious (TR4)	20.2%	27.5%
Highly suspicious (TR5)	3.9%	3.9%

**Table 3:** Prevalence of hyperfunctional nodules among those recommended for FNA by ATA, EU-TIRADS, and ACR-TIRADS, lesions and patient based for all nodules ≥ 10 mm (*n* = 1029) and only for nodules ≥ 15 mm (*n* = 545)

	All nodules			For nodules ≥ 15 mm		
	ATA	EU-TIRADS	ACR-TIRADS	ATA	EU-TIRADS	ACR-TIRADS
Lesions-based	6.4% (33/514)	6.9% (22/319)	6.5% (13/199)	7.2% (29/401)	7.6% (21/277)	7.5% (13/174)
Prevalence of hyperfunctional nodules under those recommended for FNA						
Patient-based	8.9% (33/370)	8.6% (22/257)	7.6% (13/172)	9.4% (29/310)	9.3% (21/227)	8.4% (13/154)
Proportion of patients with at least one nodule recommended for FNA being hyperfunctional among those patients with nodules recommended for FNA						

The incidence of hyperfunctional nodules was not significantly different between female (9.5%) and male (6.1%) patients (odds ratio, 1.61; 95% CI, 0.81 to 3.17, *p* = 0.171). However, the median age of 59.66 (Q1, 49.28; Q3, 65.83) years in patient with hyperfunctional nodules was higher than the median age of 52.86 (Q1, 43.02; Q3, 63.70) years in patients without hyperfunctional nodules (*p* = 0.011). Median TSH values differed between patients with (1.10 μU/ml; Q1, 0.87; Q3, 1.27) and without hyperfunctional nodules (1.23 μU/ml; Q1, 0.87; Q3, 1.88) (*p* = 0.028).

Median diameter of nodules was 20.0 mm for scintigraphically hypofunctional, 13.0 mm for indeterminate, and 17.0 mm for hyperfunctional nodules with a statistical significance between the groups (Kruskal-Wallis *H* test; *χ*^2^(2) = 154.03; *p* < 0.001). Pairwise comparison (Dunn’s procedure) demonstrated a difference in the diameter of indeterminate to hypofunctional nodules (*p* < 0.001) and indeterminate to hyperfunctional nodules (*p* < 0.001). No difference was found between the diameter of hypofunctional and hyperfunctional nodules (*p* = 0.60).

Association of sonographic features and functional status is described in Table 4 and association of functional status and sonographic classification systems in Table 5.

**Table 4:** Association of sonographic features and functional status. Numbers in brackets are 95% confidence intervals

Parameter	Number of cold or indeterminate nodules	Number of hot nodules	Odds ratio	*p* value
Diameter				*< 0.004*
< 15 mm	471	13 (2.9%)	1	
≥ 15 mm	507	38 (7.0%)	2.682 (1.381–5.208)	
Composition				0.670
Solid	471	22 (4.5%)	1	
Mixed	507	29 (5.4%)	1.139 (0.625–2.076)	
Echogenicity				0.500
Iso- or hyperechogenic	747	37 (4.7%)	1	
Hypoechogenic	231	14 (5.7%)	1.257 (0.646–2.446)	
Shape				0.715
Wider than tall	950	50 (5.0%)	1	
Taller than wide	28	1 (3.4%)	0.682 (0.086–5.383)	
Margin				0.572
Ill-defined or lobulated	61	2 (3.2%)	1	
Smooth	917	49 (5.1%)	1.534 (0.347–6.785)	
Hyperechoic foci				0.493
None	888	45 (4.8%)	1	
Calcifications or comet tails	90	6 (6.2%)	1.381 (0.548–3.476)	

Statistically significant findings, i.e. *p* < 0.05, were emphsised in italic

**Table 5:** Association of functional status and sonographic classification systems. Numbers in brackets are 95% confidence intervals

Parameter	Number of cold or indeterminate nodules	Number of hot nodules	Odds ratio	*p* value
ATA category				0.052
Benign—low suspicious	786	35 (4.3%)	1	
Intermediate and high suspicious	192	16 (7.7%)	1.905 (0.995–3.648)	
FNA required according to ATA				*0.034*
No FNA	497	18 (3.5%)	1	
FNA	481	33 (6.4%)	1.944 (1.051–3.596)	
ACR-TIRADS score				0.181
0–3	757	35 (4.4%)	1	
≥ 4	221	16 (6.8%)	1.549 (0.815–2.945)	
ACR-TIRADS FNA				0.340
No FNA	792	38 (4.6%)	1	
FNA	186	13 (6.5%)	1.394 (0.704–2.759)	
EU-TIRADS category				0.124
Benign and low risk	763	35 (4.4%)	1	
Intermediate and high risk	215	16 (6.9%)	1.661 (0.869–3.173)	
EU-TIRADS FNA				0.076
No FNA	681	29 (4.1%)	1	
FNA	297	22 (6.9%)	1.721 (0.945–3.136)	

Statistically significant findings, i.e. *p* < 0.05, were emphsised in italic

### Results of fine needle aspiration cytology and histology

Fine needle aspiration cytology was available for 128 hypofunctional or indeterminate nodules (out of 1029) in 114 patients but not for hyperfunctional nodules. Of those, 21 were Thy1, 81 Thy 2, 25 Thy 3, 0 Thy 4, and 1 Thy 5 according to the Royal College of Pathologist Thyroid Cytology Specimens Reporting System. Pathology results were available for 169 hypofunctional or indeterminate nodules in 83 patients (156 benign, 11 papillary thyroid carcinoma, 1 follicular thyroid carcinoma, 1 medullary carcinoma) and 3 hyperfunctional nodules (out of 51) in 3 patients (3 benign).

## Discussion

**Fig. 5:**
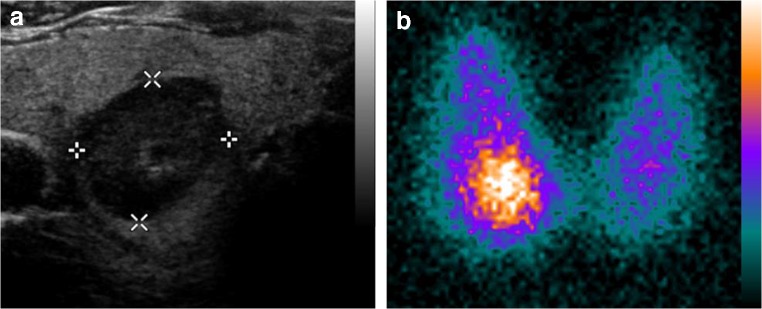
**a** Patient with two hypoechoic nodules in the right thyroid lobe—one in the upper half with a maximum diameter of 17 mm (not shown) and the other one in the lower half with a maximum diameter of 19 mm. Both nodules would require fine needle aspiration cytology according to ATA 2015, EU-TIRADS, and ACR-TIRADS. **b**^99m^Tc-pertechnetate scintigraphy of the same patient, revealing the lower nodule to be hyperfunctional, despite having a TSH value within reference range (1.02 μU/ml). The upper nodule was classified as indeterminate. The patient declined FNA of the scintigraphically indeterminate nodule and opted for diagnostic hemithyroidectomy. Histologic workup demonstrated both nodules to be benign

In this study, we investigated a large cohort of euthyroid patients who presented for the workup of thyroid nodules > 10 mm and were assessed with both sonography and thyroid scintigraphy with ^99m^Tc-pertechnetate according to the German guidelines [21] . Interestingly, nodules in need of FNA according to ATA 2015, EU-TIRADS, or ACR-TIRADS appeared hyperfunctional on thyroid scintigraphy in as many as 8.9%, 8.6%, and 7.6% of patients considering all nodules ≥ 10 mm and 9.4%, 9.3%, and 8.4% of patients considering only nodules ≥ 15 mm (Fig. 5). Patients with hyperfunctional nodules had lower TSH values compared with patients without hyperfunctional nodules independent of gender. No association between sonographic patterns and nodule functional status in scintigraphy was found.

Multiple studies have already investigated cohorts of patients presenting with hyperfunctional nodules on thyroid scintigraphy [23–26]. Treglia et al. summarized these findings in their meta-analysis showing 50% of those patients to have normal TSH values [14]. Our findings of hyperfunctional nodules occurring in a population of patients with normal TSH values are in line with those findings. However, the data of our analysis for the first time allow an estimation of the prevalence of hyperfunctional nodules in euthyroid patients presenting for workup of nodular goiter, which was found as high as 8.9% (or even 9.4% considering only nodules ≥ 15 mm) in our cohort. Schenke et al. have previously investigated how hyperfunctional thyroid nodules are classified by TIRADS according to Kwak [19] in a mixed population with normal or suppressed TSH values and found 64.8% of nodules classified as suspicious (Kwak-TIRADS 4b or higher). In our euthyroid patient group, there was a significant difference in ultrasound-based nodule classification between different classification systems with FNA recommended for 65.7% (ATA 2015), 43.1% (EU-TIRADS), and 25.5% (ACR-TIRADS) of hyperfunctional nodules.

Patients with hyperfunctional thyroid nodules had a slightly lower median TSH value than patients without hyperfunctional nodules. However, while the difference in median TSH of 1.10 μU/ml compared with 1.23 μU/ml was statistically significant, it is probably too small to be of clinical relevance. This finding confirms previous studies which have shown TSH to be insufficient to rule out autonomously functional nodules [15]. In our study, only nodule size was correlated with hyperfunctionality, whereas no correlation of sonographic patterns (as assessed by ATA 2015, EU-TIRADS, and ACR-TIRADS) and functional status as assessed by scintigraphy was found, emphasizing that scintigraphy provides complementary diagnostic information to sonography.

To our knowledge, this is the first study demonstrating a prevalence of up to 6.9% (or even 7.6% for nodules ≥ 15 mm) of hyperfunctional nodules among those nodules for which FNA is recommended by different ultrasound-based classification systems in euthyroid patients. Hyperfunctional nodules are generally assumed to be almost exclusively benign, a notion also reflected in current guidelines such as the American Thyroid Association’s [4, 11–13]. Therefore, thyroid scintigraphy provides complementary diagnostic information to ultrasound not only in hyperthyroid but also in euthyroid patients. It should also be considered to adjust the different national recommendations for the use of thyroid scintigraphy—e.g., in the BTA guidelines, thyroid scintigraphy is not mentioned at all as a tool to further investigate thyroid nodules. However, regional differences in environmental factors—such as iodine replete vs. iodine-deficient regions—should be considered.

It is also important to keep in mind that, while uncommon, malignancy in toxic thyroid nodules cannot be entirely excluded. Indeed, one patient with follicular thyroid cancer, cared for in our institution (not included in the study population), initially presented with a hyperfunctional nodule which turned out to be malignant. Some studies such as a review by Mirfakhraee et al. report malignancy rates of up to 3.1% for hyperfunctional nodules [27–29]. Further studies evaluating the malignancy rate of hyperfunctional nodules seem warranted.

### Limitations

Our work has several limitations—most of them due to the retrospective approach of our data analysis. Histologic or cytologic workup was only available for a minority of nodules and due to the fact of being conducted in a tertiary referral hospital there was also no systemic patient follow-up. A potential limitation could be that ^99m^Tc-pertechnetate was used as radiotracer for thyroid scintigraphy. ^99m^Tc-pertechnetate is taken up into thyrocytes by the NIS but not organified. Previous studies have shown that about 5% of nodules hyperfunctional in ^99m^Tc-pertechnetate are so-called trapping only nodules, i.e., they are cold in ^123^I scintigraphy and can potentially harbor malignancy [25]. Furthermore, this is a single-center study, in a region with a history of iodine deficiency. The prevalence of hyperfunctional nodules might be different for regions without a history of iodine deficiency. To substantiate the findings of this study, prospective multi-center trials with systematic cytological and histologic workup, scintigraphy preferably preformed with ^123^I, and a systematic follow-up are desirable.

## Conclusion

In conclusion, hyperfunctional thyroid nodules frequently occur in euthyroid patients where ultrasound-based classification schemes recommend FNA to rule out malignancy. Given the low malignancy risk of hyperfunctional nodules, thyroid scintigraphy therefore provides complementary diagnostic information to sonography and can help to reduce unnecessary FNA and—potentially—unnecessary diagnostic hemithyroidectomies. However, prospective multi-center trials with systematic cytological and histological workup, thyroid scintigraphy, and a systematic follow-up are required to assess the full incremental value of thyroid scintigraphy in the management of thyroid nodules.

## Abbreviations

ACR: American College of Radiology
ATA: American Thyroid Association
EU: European
FNA: Fine needle aspiration cytology
NIS: Sodium/iodine symporter
Tc: ^99m^Technetium
TIRADS: Thyroid Imaging Reporting and Data System
